# Identifying miRNA-mRNA regulatory relationships in breast cancer with invariant causal prediction

**DOI:** 10.1186/s12859-019-2668-x

**Published:** 2019-03-15

**Authors:** Vu VH Pham, Junpeng Zhang, Lin Liu, Buu Truong, Taosheng Xu, Trung T. Nguyen, Jiuyong Li, Thuc D. Le

**Affiliations:** 1 School of Information Technology and Mathematical Sciences, University of South Australia, Adelaide, Australia; 2 School of Engineering, Dali University, Dali, Yunnan, China; 3 Pham Ngoc Thach University of Medicine, Ho Chi Minh, Vietnam; 4 Institute of Intelligent Machines, Heifei Institutes of Physical Science, Chinese Academy of Sciences, Hefei, China

**Keywords:** Invariant prediction, Causality, Inference method, microRNA, mRNA, Regulatory relationship

## Abstract

**Background:**

microRNAs (miRNAs) regulate gene expression at the post-transcriptional level and they play an important role in various biological processes in the human body. Therefore, identifying their regulation mechanisms is essential for the diagnostics and therapeutics for a wide range of diseases. There have been a large number of researches which use gene expression profiles to resolve this problem. However, the current methods have their own limitations. Some of them only identify the correlation of miRNA and mRNA expression levels instead of the causal or regulatory relationships while others infer the causality but with a high computational complexity. To overcome these issues, in this study, we propose a method to identify miRNA-mRNA regulatory relationships in breast cancer using the invariant causal prediction. The key idea of invariant causal prediction is that the cause miRNAs of their target mRNAs are the ones which have persistent causal relationships with the target mRNAs across different environments.

**Results:**

In this research, we aim to find miRNA targets which are consistent across different breast cancer subtypes. Thus, first of all, we apply the Pam50 method to categorize BRCA samples into different "environment" groups based on different cancer subtypes. Then we use the invariant causal prediction method to find miRNA-mRNA regulatory relationships across subtypes. We validate the results with the miRNA-transfected experimental data and the results show that our method outperforms the state-of-the-art methods. In addition, we also integrate this new method with the Pearson correlation analysis method and Lasso in an ensemble method to take the advantages of these methods. We then validate the results of the ensemble method with the experimentally confirmed data and the ensemble method shows the best performance, even comparing to the proposed causal method.

**Conclusions:**

This research found miRNA targets which are consistent across different breast cancer subtypes. Further functional enrichment analysis shows that miRNAs involved in the regulatory relationships predicated by the proposed methods tend to synergistically regulate target genes, indicating the usefulness of these methods, and the identified miRNA targets could be used in the design of wet-lab experiments to discover the causes of breast cancer.

**Electronic supplementary material:**

The online version of this article (10.1186/s12859-019-2668-x) contains supplementary material, which is available to authorized users.

## Background

The human transcriptome is composed of 98% of non-coding RNAs (ncRNAs) and only 2% of protein-coding RNAs [1]. However, research into the roles of ncRNAs is still in the early stage. The emergence of ncRNAs as new key players in cancer development and progression has shifted our understanding of gene regulation [1, 2], especially since the discovery of microRNAs (miRNAs). miRNAs are short ncRNAs that regulate gene expression at the post-transcriptional level and identified as the drivers in diverse disease conditions including cancers, where they function either as oncogenes or as tumor suppressors [3, 4]. Recent years have also seen the discovery of several other types of ncRNAs, including long non-coding RNAs (lnRNAs), pseudogenes and circular RNAs (cirRNAs), along with their regulatory functions in disease conditions [4]. There also has been evidence that mRNAs, miRNAs, and other ncRNAs work in concert to regulate cancer development and progression [5, 6].

There have been several methods developed to explore miRNA functions, including those for predicting miRNA targets and regulatory modules (see [7] for a review), inferring miRNA sponge networks and modules [6, 8–10], and identifying cancer subtypes [11–13]. However, our understanding of miRNAs’ roles in regulating cancer across different subtypes thereby permitting prognosis, diagnosis, and prediction of therapy response is still very far from complete, and reliable methods for identifying miRNA-mRNA regulatory relationships in cancer are in demand.

Existing computational methods for inferring miRNA-mRNA regulatory relationships are of two major categories: sequence-based approach and expression-based approach. The former is based on complementary base pairing, site accessibility, and evolutionary conservation; and the latter relies on the negative correlation between miRNA and mRNA expression levels. The expression-based approach can be further divided into i) correlation-based approach [14–16], and ii) causal inference approach [17–19].

Each of the approaches has its own advantages and limitations. The correlation-based and regression-based approaches [14–16] are efficient for large gene expression datasets. However, correlations or associations are not causality, but miRNA-mRNA regulatory relationships are causal relationships. A strong correlation between the expression values of a miRNA and a mRNA in a dataset may be a spurious relationship, as it could be confounded by a transcription factor. On the other hand, the causal inference approach [17–19] aims to estimate the intervention effects as in gene knockdown experiments. Therefore, this approach discovers the causal relationship between miRNAs and mRNAs, i.e. the regulation of miRNAs on mRNAs directly or indirectly through other factors. As gene knockdown experiments are expensive to conduct given the large number of miRNAs and mRNAs, the causal methods can be used as an alternative to identify the regulation of miRNAs on mRNAs.

While these causal inference methods help remove spurious relationships, they have high computational complexity and therefore are not scalable to large datasets. With the fact that using proper computational facility would alleviate the problem to certain extend, we have exploited the parallel processing-technique for the causal method jointIDA by using its parallel implementation in the ParallelPC package [20] but it still consumes much time when running with large datasets. Moreover, these methods do causal inference based on the causal graphs learnt from data, which involves false discoveries when the sample size is not large enough.

We propose to infer the miRNA-mRNA regulatory relationships in breast cancer by adapting a recently developed causal inference method, invariant causal prediction (ICP) [21]. Applying the key idea of causal invariance used by ICP, the causes (miRNAs) of a mRNA are the ones that show consistent causal relationships with the mRNA across different environments. The “different environments” can be understood as different datasets obtained from different sources/labs for studying the same disease, or different types of datasets such as observational data and data obtained from intervention experiments.

In this paper, we identify miRNA-mRNA causal regulatory relationships in breast cancer with an assumption that miRNAs are causal for mRNAs when they have consistent causal relationships across cancer subtypes. We firstly apply the Pam50 method [22, 23] to the breast adenocarcinoma (BRCA) dataset of The Cancer Genome Atlas (TCGA) [24] to classify the samples into 5 different breast cancer subtypes, Basal, Her2, LumA, LumB, and Normal-like. We then use the ICP method to search for miRNA-mRNA pairs that show persistent causal relationships across different subtypes. It is shown that if the simultaneous noise interventions assumption is satisfied, i.e. if the input datasets are generated by the linear structural equation models under the simultaneous noise interventions, then the causal predictors are identifiable using the ICP method (Section 4.3 of Reference [21]). The simultaneous noise interventions are interventions which change the noise or error distributions at many variables simultaneously. A noise intervention is a type of soft intervention which “disturbs” a variable by changing its error distribution. In our application with the BRCA dataset, we have divided the dataset into multiple datasets corresponding to different environments (cancer subtypes) by the Pam50 method based on the expression of 50 mRNAs. This means that in the different cancer subtype datasets, the expressions of these 50 mRNAs are significantly different, which could be considered as the result of noise interventions in cancer subtypes at these 50 mRNAs. This indicates that the input datasets used in our study satisfies the assumption of ICP, so the findings are potentially causal. After that, we validate the predictions with miRNA transfection data, and the results show that our proposed method performs better than the existing methods that are based on correlation, regression or other causal discovery methods such as idaFast [17] or jointIDA [25]. The method is also much faster than the other existing casual discovery-based methods as the ICP method does not need to learn a complete causal graph from data (which is time consuming) whereas the existing methods do. Furthermore, the ICP does not fit a model in each environment and then do pair-wise comparison between the models. Instead, it fits a global model to all samples and calculate the residuals of each sample when fitting the global model, then compares the residual distribution in each environment.

We also develop an ensemble method that combines the proposed method with a correlation-based method (Pearson) and a regression-based method (Lasso) to take the merits of different approaches. Using experimentally confirmed databases, miRTarbase 6.1, TarBase 7.0 and miRWalk 2.0, we show that the ensemble method is the best method compared to its individual component methods, including the proposed causal invariance method.

In addition, functional enrichment analysis shows that the identified miRNA-mRNA relationships are highly enriched in functions and processes related to breast cancer, suggesting the usefulness of the method. Novel interactions identified by the proposed methods could be good candidates for follow-up wet-lab experiments to explore their roles in breast cancer.

## Results

Predicted miRNA-mRNA regulatory relationships are checked with the transfection data by using the R package miRLAB [26] and the experimentally confirmed databases as these databases are about the confirmed miRNA-mRNA interactions. For the checking with the transfection data, if for a predicted miRNA-mRNA relationship, its absolute value of the *log*_2_ fold-change in the transfection data is larger than a predefined threshold (i.e. 0.3 in our experiments), then the predicted miRNA-mRNA relationship is considered as confirmed, i.e. supported.

The transfection data is obtained from the TargetScoreData package [27] and it can be found in the Additional file 1. In the miRNA transfection experiment, the transfection data was created from 84 Gene Expression Omnibus (GEO) series [28]. The raw data is downloaded and the *log*_2_ fold-change of the expression of a mRNA in treatment (miRNA transfected) is calculated by comparing the expression levels of the mRNA between transfected and controlled samples. The higher the absolute value of the *log*_2_ fold-change is, the more significant the differential expression level of the mRNA is. For the validation with the experimentally confirmed databases, we build the ground truth by combining the information from miRTarbase version 6.1 [29], TarBase version 7.0 [30], and miRWalk version 2.0 [31]. These three databases provide experimentally validated miRNA-target interactions and they are available in the Additional file 2.

The performance of a method will be measured using the number of discovered miRNA-mRNA interactions that have been validated by using the experimentally confirmed databases or the transfection data. The higher the number of validated miRNA-mRNA interactions a method has, the better the method is.

### Comparison of results

To evaluate the performance of hiddenICP, we have used the other 4 methods in our experiments for comparison, including idaFast [17] in pcalg package [32], jointIDA_direct [25], Pearson [33] and Lasso [34]. idaFast is a function which is used to estimate total causal effect of one variable on various target variables. jointIDA estimates total joint effect of a set of variables on another variable. Pearson and Lasso estimate the correlation coefficient and the regression coefficient of two variables respectively. These methods are chosen because idaFast and jointIDA are causal methods with similar goal as ours while Pearson and Lasso are popular correlation and regression methods.

With hidden ICP, we run it in two separate scenarios. In the first scenario, we randomly divide the samples into three datasets with similar sizes, each corresponding to an environment. In the second scenario, Pam50 [22, 23] is used to categorize the samples based on different cancer subtypes, including Basal, Her2, LumA, LumB, and Normal-like, to create datasets for the different environments.

The top miRNA-mRNA interactions predicated by each of the 6 methods are selected to be checked with the transfection data and experimentally confirmed interactions. The miRNA-mRNA interactions estimated by the methods are ordered by their correlation/causal effects/scores, the larger a correlation/causal effect/score is, the higher the relationship is in the list. To have a comprehensive analysis, we select the top 500, 1000, 1500, and 2000 miRNA-mRNA interactions for the validation, and we also do the validation with respect to each miRNA by selecting the top 50, 100, 150 and 200 interactions in which the miRNA is involved.

**Fig. 1:**
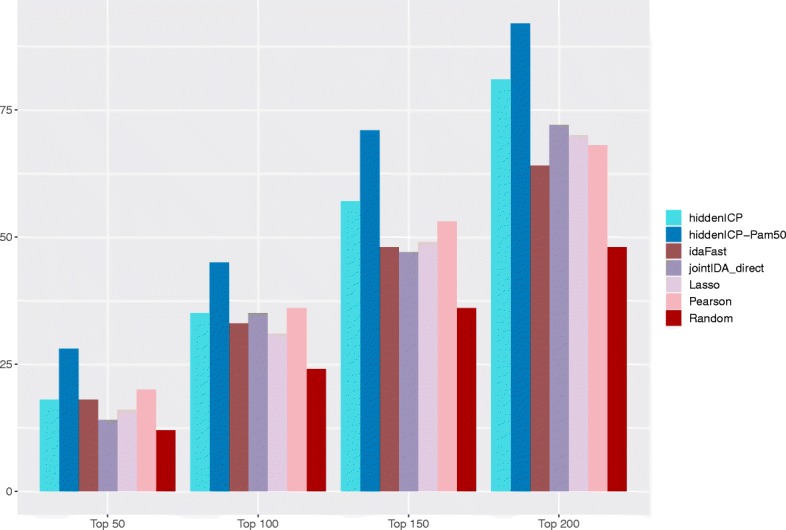
Checking using the transfection data. For each miRNA, the top 50, 100, 150 and 200 predicted miRNA-mRNA interactions are selected and checked against the transfection data. Each bar in the diagram shows the total number of supported interactions accumulated over all the miRNAs checked

First of all, we check the results of the 6 methods by using the transfection data as the ground truth. As the miRNAs in the transfection data are not complete, for this case, it is not fair to compare the top miRNA-mRNA interactions for all miRNAs. Thus, for the checking using the transfection data, we only compare the results based on the top of miRNA-mRNA interactions with respect to each of the miRNAs. The comparison result is shown in Fig. 1. In Fig. 1, besides the 6 methods, we also include the null experiment to show the superiority of these methods. In the null experiment, we pick randomly 30 miRNAs and tops k targets for each miRNA (for k=50, 100, 150, and 200) from the BRCA dataset. We run the experiment 100 times then calculate the average values and consider them as the final values. It can be seen that in all four cases with the top 50, 100, 150 and 200 “interactions predicted” for each miRNA, hiddenICP using Pam50 (hiddenICP-Pam50 in the figure) outperforms the other methods in discovering miRNA-mRNA regulation relationships. When combining with Pam50, hiddenICP (i.e. hiddenICP-Pam50) shows the best performance, indicating that the method may serve as a good tool in predicting miRNA targets. The top predicted miRNA-mRNA interactions for each miRNA by hiddenICP-Pam50 can be found in Additional file 3.

When we validate the top predicted miRNA-mRNA interactions using the experimentally confirmed databases, there is no method which finds a number of experimentally confirmed miRNA-mRNA interactions larger than other methods in all experiments with different selected top ranked interactions. For instance, with the top 500 predicted miRNA-mRNA interactions, Lasso is the best method which finds the most confirmed miRNA-mRNA interactions while Pearson and Lasso are the best in the experiment with the top 1000 predicted miRNA-mRNA interactions. When we validate the top 50 predicted miRNA-mRNA interactions for each miRNA, Pearson is the best while the performance of Lasso is even worse than the performance of idaFast. However, in most cases, Pearson and Lasso outperforms others.

**Fig. 2:**
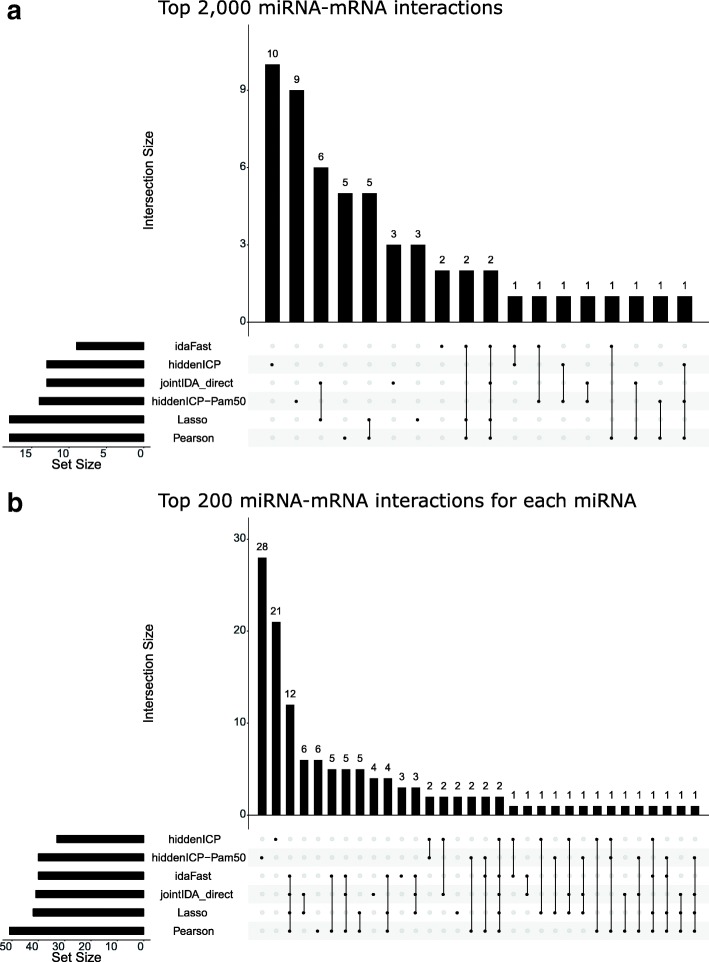
Overlap between different methods. The top miRNA-mRNA interactions validated by using the experimentally confirmed database information. **a** For each method, the figure shows that among the top 2000 predicted miRNA-mRNA interactions, how many interactions have been validated to be true by the databases (on the bottom left), and between the different methods how the validated interactions overlap with each other (the dotted lines and the diagram on top). **b** For each method, the figure shows that among the top 200 predicted miRNA-mRNA interactions for each miRNA, how many interactions have been validated to be true by the databases (on the bottom left), and between the different methods how the validated interactions overlap with each other (the dotted lines and the diagram on top)

In addition, the findings of different methods are complementary, as indicated in Fig. 2a and b. Figure 2a shows the intersection of predicted results of methods with top 2000 interactions for all miRNAs (The result of hiddenICP-Pam50 can be found in Additional file 4) while Fig. 2b shows the intersection of predicted results of methods with top 200 interactions for each miRNA. It can be seen that in some cases such as top 2000 interactions for all miRNAs and top 200 interactions for each miRNA in this figure, although Pearson and Lasso detect more confirmed miRNA-mRNA interactions, others could discover some interactions which cannot be identified by Pearson and Lasso. Thus, to take the advantages of Pearson, Lasso, and other methods, we introduce an ensemble method which combines Pearson, Lasso, and other methods to predict miRNA-mRNA regulatory relationships in the next section.

**Hidden ICP forms a good performance in identifying miRNA-mRNA regulatory relationships of ensemble method**

**Fig. 3:**
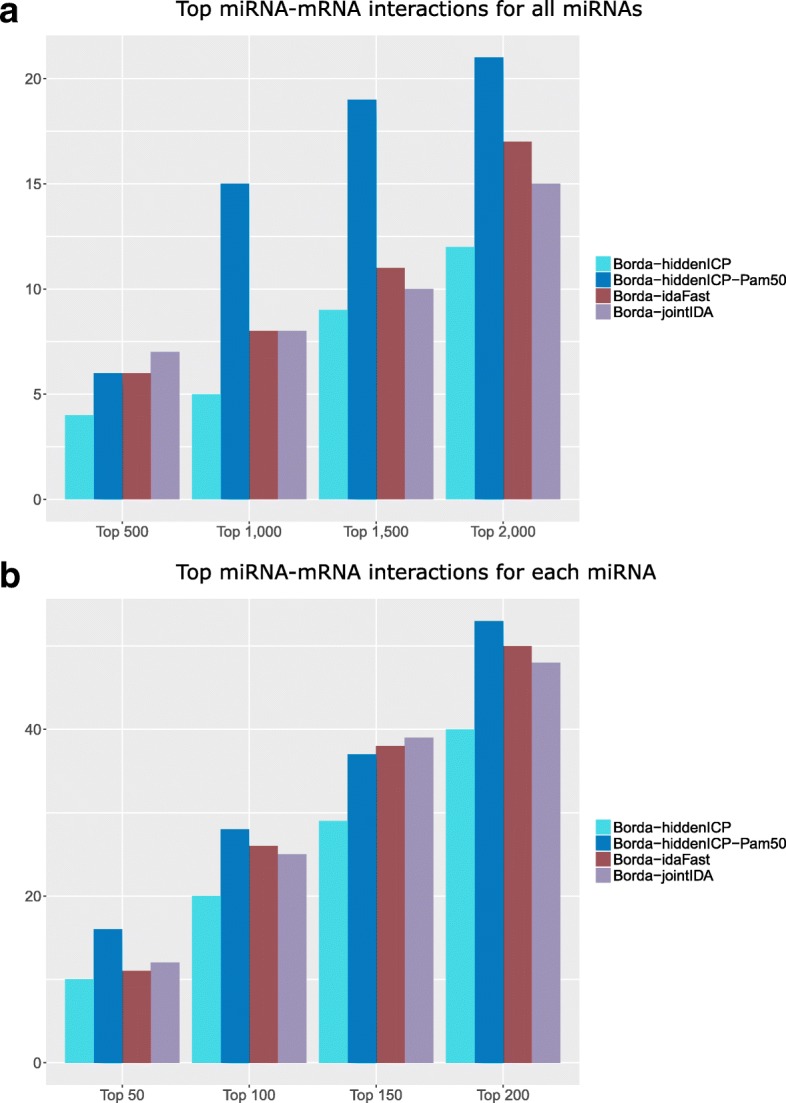
Validation using the experimentally confirmed databases. The compared methods are the Borda function which integrate Pearson and Lasso with hiddenICP, hiddenICP-Pam50, idaFast, or jointIDA. **a** The top 500, 1000, 1500 and 2000 predicted miRNA-mRNA interactions for all miRNAs are selected and validated against the experimentally confirmed databases. Each bar in the diagram shows the total number of validated interactions of all miRNAs. **b** For each miRNA, the top 50, 100, 150 and 200 predicted miRNA-mRNA interactions are selected and validated against the experimentally confirmed databases. Each bar in the diagram shows the total number of validated interactions accumulated over all the miRNAs validated

Based on the observations that different methods may provide complementary findings of miRNA-mRNA interactions, and Pearson and Lasso individually may perform better than the other methods, we use the Borda function in the package miRLAB [26] to integrate Pearson [33], Lasso [34] with others (hiddenICP, hiddenICP-Pam50, idaFast, jointIDA) to generate ensembles for predicting miRNA-mRNA interactions. This ensemble method Borda will get the average of the rankings from individual methods. The validation results of the ensembles are shown in Fig. 3a and b, for the validation of the collection of top interactions of all miRNAs and the validation of the top interactions around individual miRNAs, respectively. In both cases, the Borda with Pearson, Lasso and hiddenICP using Pam50 outperforms others.

## Discussion

### miRNAs tend to synergistically regulate target genes

In this section, we focus on studying miRNA synergism based on the top 50, 100, 150 and 200 target genes for each miRNA identified by hiddenICP-Pam50. For each possible miRNA synergistic pair *miRNA*_*i*_ and *miRNA*_*j*_, *i*≠*j*, the hypergeometric test is used to evaluate the significance of the shared mRNAs by these two miRNAs. The significance *p*-value is calculated as follows:

1 $$\begin{array}{@{}rcl@{}}  &p=1-\sum\limits_{x=0}^{n-1} \frac{{K \choose x} {N-K \choose M-x}}{{N \choose M}}, \end{array} $$

where *N* denotes the number of all mRNAs of interest, *K* is the number of mRNAs interacting with miRNA_*i*_, *M* is the number of mRNAs interacting with miRNA_*j*_, *n* is the number of the shared mRNAs by miRNA_*i*_ and miRNA_*j*_

**Fig. 4:**
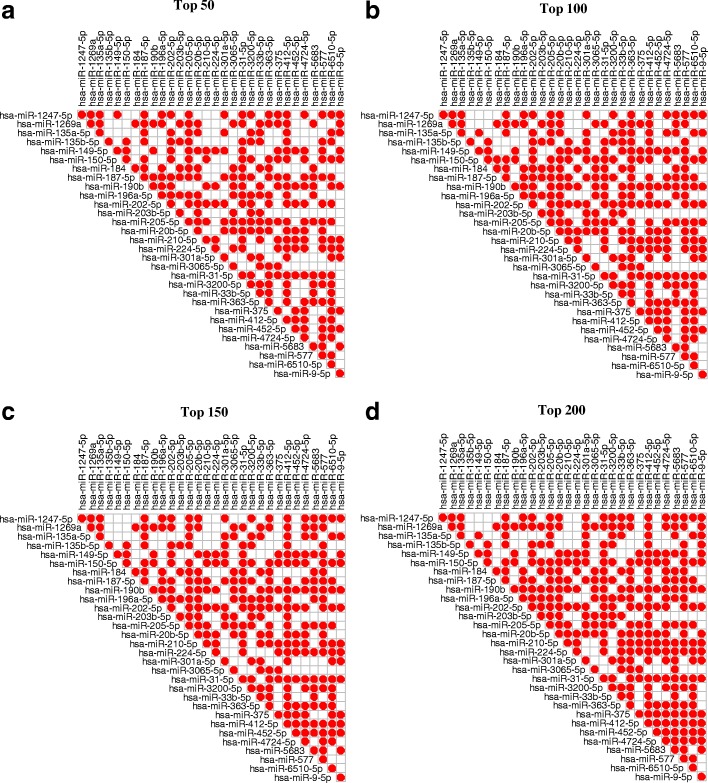
Heatmap of miRNA-miRNA synergistic relationships. Relationships in the top 50 (**a**), 100 (**b**), 150 (**c**) and 200 (**d**) target genes for each miRNA identified by hiddenICP-Pam50. A red dot indicates that there is a synergistic relationship between two miRNAs

The miRNA-miRNA pairs with significant sharing of mRNAs (e.g. *p*-value <0.05) are regarded as miRNA-miRNA synergistic pair. We set the *p*-value cutoff as 0.05 (adjusted by Benjamini & Hochberg method). As shown in Fig. 4, each miRNA tends to synergistically regulate target genes with at least one other miRNA. In terms of its top 50, 100, 150 and 200 target genes, each miRNA synergistically regulates target genes with at least 9, 11, 10 or 11 other miRNAs, respectively. This result indicates that miRNAs may involve in many biological processes by synergistically regulating target genes.

### Several miRNAs are significantly enriched in functions or diseases related to BRCA

In this section, we conduct GO [35], KEGG [36], Reactome [37] and DisGeNET [38] enrichment analysis of top target genes for each miRNA identified by hiddenICP-Pam50. The functional enrichment analysis of the top target genes for each miRNA identified by hiddenICP-Pam50 is not for the purpose of comparing different methods. The analysis is to provide an evidence to suggest the usefulness of the method in breast cancer research. Thus, among the four cases (top 50, 100, 150 and 200 interactions for each miRNA) in the “Comparison of results” section, we only used the top 50 interactions for each miRNA for enrichment analysis. In Table 1, out of the 30 miRNAs, 12, 10, 13 and 18 miRNAs are significantly associated with at least one GO, KEGG, Reactome and DisGeNET terms, respectively. As shown in Table 2, several miRNAs are significantly enriched in functions or diseases related to BRCA. The results show that the findings using our methods are biologically meaningful in the BRCA dataset. The detailed enrichment analysis results can be seen in Additional file 5.

**Table 1:** Functional enrichment analysis of the top 50 target genes for each miRNA identified by hiddenICP-Pam50 (at least one term more than 0)

miRNAs	#GO terms	#KEGG terms	#Reactome terms	#DisGeNET terms
hsa-miR-187-5p	3	0	0	0
hsa-miR-1269a	7	0	0	0
hsa-miR-184	0	1	0	0
hsa-miR-205-5p	23	0	2	147
hsa-miR-196a-5p	7	8	0	58
hsa-miR-203b-5p	2	1	2	0
hsa-miR-375	0	2	5	23
hsa-miR-5683	33	0	0	54
hsa-miR-6510-5p	0	1	11	0
hsa-miR-20b-5p	2	0	0	1
hsa-miR-363-5p	51	1	4	56
hsa-miR-577	0	0	2	38
hsa-miR-135b-5p	27	0	0	26
hsa-miR-150-5p	17	0	3	32
hsa-miR-412-5p	0	0	0	25
hsa-miR-1247-5p	40	0	15	8
hsa-miR-4724-5p	0	0	5	0
hsa-miR-3065-5p	0	0	0	4
hsa-miR-31-5p	1	2	0	1
hsa-miR-135a-5p	0	0	2	2
hsa-miR-9-5p	0	2	5	32
hsa-miR-3200-5p	0	1	0	21
hsa-miR-33b-5p	0	0	4	0
hsa-miR-202-5p	0	5	0	39
hsa-miR-301a-5p	0	0	3	6

**Table 2:** Several miRNAs are significantly enriched in functions or diseases related to BRCA

miRNAs	Functions or diseases associated with BRCA	Enriched terms
hsa-miR-187-5p	regulation of mononuclear cell migration	GO:0071675
hsa-miR-205-5p	negative regulation of cell fate commitment	GO:0010454
	regulation of cell fate specification	GO:0042659
	mesodermal cell fate specification	GO:0007501
	endodermal cell fate commitment	GO:0001711
	mesodermal cell fate commitment	GO:0001710
	Sporadic Breast Carcinoma	umls:C1336076
hsa-miR-196a-5p	Rap1 signalling pathway	hsa04015
hsa-miR-5683	negative regulation of mesenchymal cell apoptotic	GO:2001054
	regulation of mesenchymal cell apoptotic process	GO:2001053
	mesenchymal cell apoptotic process	GO:0097152
	endodermal cell fate commitment	GO:0001711
	regulation of neural precursor cell proliferation	GO:2000177
hsa-miR-363-5p	mononuclear cell migration	GO:0071674
	positive regulation of mononuclear cell migration	GO:0071677
	epidermal cell differentiation	GO:0009913
	IL-17 signalling pathway	hsa04657
hsa-miR-577	Sporadic Breast Carcinoma	umls:C1336076
hsa-miR-135b-5p	Inflammatory Breast Carcinoma	umls:C0278601
hsa-miR-1247-5p	epidermal cell differentiation	GO:0009913
hsa-miR-202-5p	IL-17 signalling pathway	hsa04657

Besides hiddenICP-Pam50, other methods may also identify some miRNAs that are enriched for breast cancer related pathways or functional terms. However, this analysis is not for the comparison between methods. The purpose of the functional enrichment analysis of hiddenICP-Pam50 is to provide an evidence to suggest the usefulness of the method in breast cancer research.

### Identifying miRNA-mRNA regulatory relationships in cancer subtypes

As each cancer includes several subtypes and each subtype has different characteristics, a miRNA-mRNA regulatory relationship in a cancer subtype might not necessarily exist in other cancer subtypes. The ICP method aims to find the miRNA-mRNA relationships which persistently exist across different environments or cancer subtypes, thus the miRNA-mRNA regulatory relationships which are specific to a cancer subtype may not be discovered by the method.

## Conclusions

From the fact that miRNAs regulate gene expression by binding the 3’-UTR of mRNAs at the post-transcriptional level [6, 39–41], they are important in various biological processes in the human body and identifying their regulation mechanisms plays a salient role in diagnostics and therapeutics for a wide range of diseases. At the present, although numerous studies have developed methods to identify the relationships of miRNAs and mRNAs, most of them detect the correlations between the expression levels of miRNAs and mRNAs while the methods discovering the cause-effect relationship have a high computational complexity. To deal with this problem, we introduce the methods to identify causal effects of miRNAs on mRNAs based on ICP [21].

ICP is a method which is used to infer causality of variables across different environments such as different datasets obtained from different sources/labs for studying the same disease or different types of datasets (observational data and data obtained from intervention experiments), and it is based on the invariance assumption of the causal relationships across different settings. The method has been designed with high dimensional data in mind and has an extension for hidden variables. These features have made the ICP method a great candidate for dealing with biological problems, where the datasets (such as gene expression data) may contain measurements of thousand of variables while some variables are hidden/unobservable.

For our method, first of all, we select top miRNAs and mRNAs with the most different median absolute deviation from BRCA dataset. We then apply Pam50 method to categorize BRCA samples into different environment settings based on different cancer subtypes. After that, we use the invariant causal prediction to find miRNA-mRNA regulatory relationships across subtypes. We validate the results with the miRNA-transfected experimental data and the results show that our method outperforms others. Moreover, to take the advantages of hiddenICP as well as Pearson and Lasso, we combine them into the ensemble method using Borda election to discover miRNA-mRNA regulatory relationships. We validate the results with the experimentally confirmed data and it shows that the ensemble method with hiddenICP-Pam50 outperforms other methods in finding the interactions and can complement to other methods in finding miRNA-mRNA interactions. Further enrichment analysis indicates that miRNAs involved in the predicted regulatory relationships tend to synergistically regulate target genes, indicating the usefulness of our methods in uncovering miRNA regulation mechanisms.

## Methods

### Overview

**Fig. 5:**
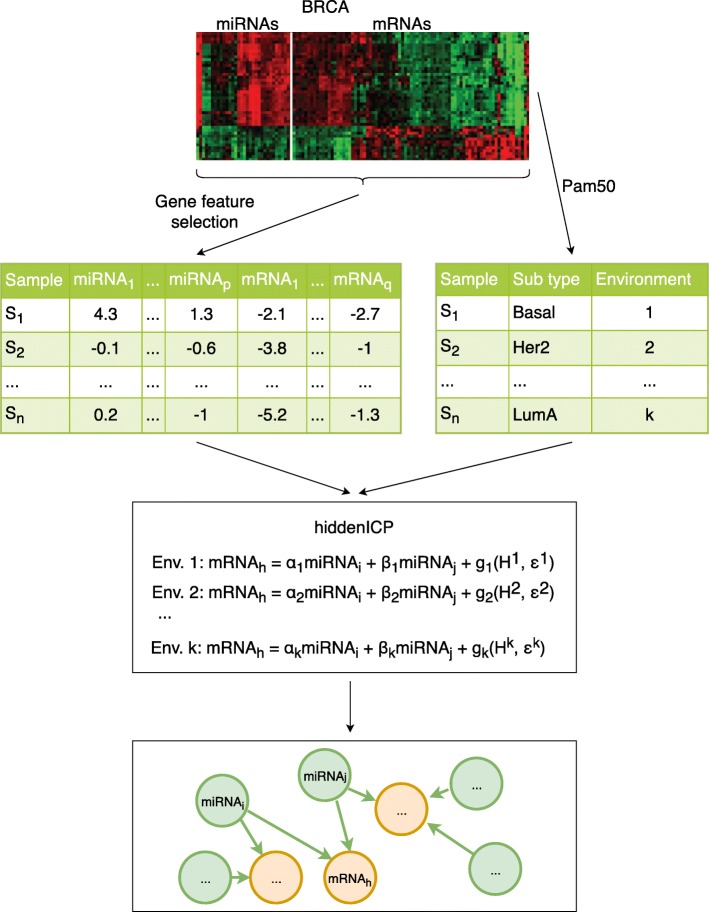
The overview of our method. The method includes three main steps, i) Select miRNAs and mRNAs with most expression variability (the gene expression is shown in the above table), ii) Categorize samples into different experiment settings and iii) Predict causal effects of miRNAs on mRNAs

The overview of our method is in Fig. 5. It has three main steps, including selecting miRNAs and mRNAs with most expression variability, categorizing samples into different experiment settings and predicting causal effects of miRNAs on mRNAs. The detail of the method is described in the following sections.

### Procedure of identifying miRNA-mRNA regulatory relationships in cancer using hidden invariant causal prediction

The algorithm for detecting miRNA-mRNA relationships includes three steps as the followings.

**Step 1**: Select miRNAs and mRNAs with most expression variability. The matched miRNA and mRNA expression samples are extracted from the BRCA dataset of TCGA [24]. In total 503 samples with matched miRNA and mRNA expression are obtained and stored in Additional file 6. Then we use the FSbyMAD function of the CancerSubtypes package [11] to select miRNAs and mRNAs with the most different Median Absolute Deviation (MAD). We select the top 30 miRNAs and top 1500 mRNAs for our experiments so that other causal inference methods including jointIDA [25] and IDA [17] could produce the results within a week for the purpose of comparison.

**Step 2**: Categorize samples into different experiment settings based on cancer subtypes by using Pam50 [22, 23] to discover miRNA targets across cancer subtypes. After the categorization, we have 107 samples in Basal subtype, 75 samples in Her2 subtype, 147 samples in LumA subtype, 116 samples in LumB subtype, and 58 samples in Normal-like subtype.

**Step 3**: Estimate the causal relationships of miRNAs on mRNAs by estimating the causal relationships of miRNAs on each mRNA through the hiddenICP function of the InvariantCausalPrediction package [21]. The detail of this step is as the following.

**Invariant causal prediction**

The ICP method considers that the causal relationship between the target and each of its direct causes maintains invariant across different environments. Based on this causal invariance idea, ICP aims to find the complete set of parents (direct causes) of the target variable by searching for the subset of predictors such that in different environments, given this subset of predictors, the conditional probabilities of the target are the same. Below are the details of the method.

We use the similar notation as that in [21]. Let $\mathcal {E}$ be the set of environments. For an environment $e \in \mathcal {E}$, (*X*^*e*^,*Y*^*e*^) is an independent and identically distributed (i.i.d.) sample in *e* where *X*^*e*^ is the set of predictor variables and *Y*^*e*^ is the target variable. *X*^*e*^ has *p* elements and $X^{e} \in \mathbb {R}^{p}$, and $Y^{e} \in \mathbb {R}$. Let $X^{e}_{S^{*}}\subseteq X^{e}$ be the subset of causal predictor variables or direct causes of *Y*^*e*^, where *S*^∗^⊆{1,…,*p*} is the indices of the predictor variables, then ICP assumes the following condition holds $\forall e \in \mathcal {E}:$

2 $$\begin{array}{@{}rcl@{}}  &X^{e} \quad \textrm{has an arbitrary distribution}, \end{array} $$

3 $$\begin{array}{@{}rcl@{}}  &Y^{e} = \mu + X^{e}\gamma^{*} + \varepsilon^{e}, \quad \varepsilon^{e} \sim F_{\varepsilon} \quad \text{and} \quad \varepsilon^{e} \perp\perp X^{e}_{S^{*}}, \end{array} $$

where *μ* is a constant intercept term, *γ*^∗^≠0, i.e. the non-zero coefficients indicating the support of the predictor variables, and *ε*^*e*^ is the error with the same distribution *F*_*ε*_ across all $e \in \mathcal {E}$

In our problem, *X* stands for miRNAs and *Y* stands for a mRNA. We apply ICP [21] to estimate causes miRNAs of a mRNA with the input data being the expression of the miRNAs and mRNA in different environments. Firstly, the Pam50 method is used to categorize the dataset into different subgroups with different cancer subtypes. Each cancer subtype is considered as an environment *e*. To increase the processing speed, instead of fitting a model for each environment, one global model is fitted for all data of all environments and the method compares the distribution of the residuals (errors) in each environment. In general, ICP loops with all subsets of predictors (miRNAs) and compares the distribution of the residuals of one environment with the other environments as a whole. If the mean and variances of residuals are the same in these environments, these subsets of predictors are potential predictors of the target. The final predictors of the target will be the intersection of these potential predictors. The detail of the ICP is described in the following steps:

1. For each *S*⊆{1,...,*p*} and $e \in \mathcal {E}:$
  Use the set S of indices of variables and fit a linear regression model for all data to have an estimated optimal coefficients $\hat {\beta }^{pred}(S)$. Let $R = Y - X\hat {\beta }^{pred}(S)$.
  Let *I*_*e*_ be the set of samples of *e* (*n*_*e*_=|*I*_*e*_|) and *I*_−*e*_ be the set of samples which are not in *e* (*n*_−*e*_=|*I*_−*e*_|). Test the null hypothesis that the mean of R is the same by using the two-sample t-test for residuals in *I*_*e*_ and *I*_−*e*_. In addition, use the F-test to test if the variances of R are the same in *I*_*e*_ and *I*_−*e*_.
2. Construct the estimator: $\hat {S}(\mathcal {E}) := \bigcap _{\text {S: not rejected}} S$.
3. Estimate the confidence set for the estimator based on the confidence of $\hat {\beta }^{pred}(S)$.

**Hidden invariant causal prediction**

ICP has an extension for hidden variables. The hidden ICP assumes that $\forall e \in \mathcal {E}:$

4 $$\begin{array}{@{}rcl@{}}  &X^{e} \quad \textrm{has an arbitrary distribution}, \end{array} $$

5 $$\begin{array}{@{}rcl@{}}  &Y^{e} = X^{e}\gamma^{*} + g(H^{e}, \varepsilon^{e}), \end{array} $$

where *H* are hidden variables, $\gamma ^{*} \in \mathbb {R}^{p}$ are causal coefficients and $g: \mathbb {R}^{q} \times \mathbb {R} \to \mathbb {R}$ is a function

In this work, we propose to apply hidden ICP to discover miRNA-mRNA regulatory relationships. This choice (instead of normal ICP) is based on the fact that in the data preparation step, we only select miRNAs and mRNAs with most expression variability as the input of ICP. Therefore in the corresponding dataset, there might be hidden miRNAs which are regulators of mRNAs. In our application of hidden ICP, the set of miRNAs with most expression variability are considered as the predictor variables. Then for each mRNA (the target or response variable), hidden ICP is used to find the direct causes, i.e. the miRNAs which regulate of the mRNA. In addition, we use Pam50 [22, 23] to categorize the samples into different subtypes, and consider the subtypes as the environments used in hidden ICP.

### Implementation

The above algorithm has been implemented and integrated into the R package miRLAB [26]. In addition, the R scripts for reproducing the results of experiments in this paper are also available upon request.

### Functional annotation of miRNAs

We do enrichment analysis for miRNA targets to annotate biological functions of miRNAs. We apply GO [35], KEGG [36], Reactome [37] and DisGeNET [38] for the top target genes based on the point estimator for the causal effects of each miRNA identified by hiddenICP using Pam50 (hiddenICP-Pam50). Since the enrichment analysis results of hundreds of target genes are too general to gain biological insight, we only focus on the enrichment analysis of the top 50 target genes for each miRNA.

## Additional files

Additional file 1 The transfection data for checking the predicted results of miRNA-mRNA regulation relationships. This file should be viewed by R. (RDA 29,853 kb)

Additional file 2 The confirmed miRNA-mRNA interactions retrieved from miRTarbase 6.1, TarBase 7.0, miRWalk 2.0. (CSV 10,149 kb)

Additional file 3 Top 50, 100, 150 and 200 predicted miRNA-mRNA interactions for each miRNA by hiddenICP-Pam50. (XLSX 414 kb)

Additional file 4 Top 2000 predicted miRNA-mRNA interactions for all miRNAs by hiddenICP-Pam50. (CSV 77 kb)

Additional file 5 The detailed enrichment analysis results of functional annotation of miRNAs. (XLSX 100 kb)

Additional file 6 The expression of matched miRNAs and mRNAs of the breast adenocarcinoma (BRCA) data set is downloaded from The Cancer Genome Atlas (TCGA). This file should be viewed by R. (RDATA 92,179 kb)

## Abbreviations

miRNAs: microRNAs
ncRNAs: Non-coding RNAs
lnRNAs: Long non-coding RNAs
cirRNAs: Circular RNAs
ICP: Invariant causal prediction
BRCA: Breast adenocarcinoma
TCGA: The Cancer Genome Atlas
MAD: Median absolute deviation
i.i.d.: Independent and identically distributed
